# Pelvic compression garments alter running biomechanics, perceived support, and fear of symptoms in postpartum women with pelvic floor dysfunction: preliminary observations from an exploratory, randomised, repeated-measures crossover design

**DOI:** 10.3389/fspor.2025.1691794

**Published:** 2026-01-09

**Authors:** Gráinne M. Donnelly, Celeste E. Coltman, Rebecca Straker, Hans von Lieres Und Wilkau, Carly L. Brantner, Isabel S. Moore

**Affiliations:** 1 Cardiff School of Sport and Health Sciences, Cardiff Metropolitan University, Cardiff, United Kingdom; 2 Pelvic, Obstetric and Gynaecological Physiotherapy, Lichfield, United Kingdom; 3 Research Institute for Sport and Exercise (RISE), Faculty of Health, University of Canberra, Canberra, ACT, Australia; 4 Intensive Rehabilitation Unit, UKSI, Bisham Abbey National Sports Centre, Nr Marlow, Buckinghamshire, United Kingdom; 5 Red Bull Athlete Performance Center, Thalgau, Austria; 6 Department of Biostatistics & Bioinformatics, Duke University, Durham, NC, United States; 7 Duke Clinical Research Institute, Durham, NC, United States

**Keywords:** sports apparel, incontinence (female), pelvic organ prolapse, postnatal, adjunct, biopsychosocial, fear of movement, physical activity

## Abstract

**Introduction:**

Pelvic compression garments are an emerging adjunct in the management of pelvic floor dysfunction (PFD), yet research investigating their efficacy and mechanism of action is limited, especially in the context of returning to running postpartum. Proposed theories for how pelvic compression garments assist postpartum women during running include alterations to running biomechanics, changes in perception, and improved pelvic floor support. It is also theorised that these factors could be influenced by levator hiatus distensibility.

**Methods:**

An exploratory, randomised, repeated-measures crossover design recruited 13 postpartum runners with self-reported symptoms of PFD. Participants' pelvic floor function and structural support were assessed. Each participant underwent two 7-min self-paced running trials, in randomised order, wearing their own shorts (control) and a pelvic compression garment (experimental) while biomechanical and accelerometer data were collected. Perceptual data on pelvic support and perceived symptoms were recorded following each running trial.

**Results:**

The pelvic compression garment significantly decreased the peak pelvic jerk, low-frequency pelvic shock attenuation, and the area under the peak pelvic acceleration curve. A significant decrease in left pelvic rotation excursion and an increase in axial trunk-to-pelvis rotation were also identified during late stance. The pelvic compression garment significantly increased perceived pelvic floor and core support and reduced fear of experiencing pelvic floor symptoms. No differences were observed between conditions for self-reported symptom experience following the running trials. Levator hiatus distensibility did not significantly interact with any biomechanical or perceptual variables.

**Discussion:**

Wearing a pelvic compression garment appears to alter running biomechanics in postpartum women with PFD in a way that produces a smoother running gait and restricts transverse pelvis motion, promoting trunk–pelvis coordination similar to that of healthy runners. In addition, wearing a pelvic compression garment increases perceived core and pelvic floor support and decreases fear of experiencing PFD symptoms compared to a control condition. Levator hiatus distensibility does not appear to interact with how symptomatic postpartum women respond to wearing a pelvic compression garment and therefore offers limited predictive value. Future studies with higher statistical power are needed to further investigate the biopsychosocial effect of pelvic compression garments.

## Introduction

Pelvic floor dysfunction (PFD) encompasses several disorders, including bladder and bowel incontinence, pelvic organ prolapse (POP), constipation, pelvic pain, and sexual dysfunction (1). Risk factors for PFD include aging, obesity, pregnancy, and childbirth (2), and low energy availability has also been identified as contributing to urinary incontinence (3). PFD is highly prevalent in postpartum women, with multiple disorders often occurring concurrently (4). For example, approximately 33% of postpartum runners experience stress urinary incontinence (SUI) and 32% report vaginal heaviness, a symptom indicative of POP (5). Vaginal heaviness and fear of movement have been specifically identified as barriers to returning to running postpartum (5). Therefore, symptoms of PFD can negatively impact quality of life of postpartum women and their ability to return to higher-impact activities such as running (6, 7).

The mechanisms associated with running-related PFD are not well understood. The pelvis plays a crucial role in transferring ground reaction forces from the lower limb to the trunk. However, research exploring this relationship found no association between ground reaction forces and surrogate measures of pelvic loading (e.g., acceleration) or pelvic shock attenuation in postpartum runners with PFD (8). Despite this, pelvic-related measures explained a higher variance in PFD than ground reaction forces (8). Recently, researchers identified that female runners (nulliparous and parous) with SUI demonstrate altered absorption and shock attenuation patterns at the hip and ankle compared to continent controls (9). In addition, among pre- to postpartum runners, differences in trunk and pelvic motion have been observed. Specifically, postpartum runners exhibit restricted trunk and pelvic motion (10, 11) and reduced hip and trunk strength (11). However, it remains unclear if and how these differences influence PFD.

While pelvic floor muscle training (PFMT) is established as the first-line management for PFD (12, 13), conservative approaches also include the use of adjunct products (14). For example, pelvic compression garments are an emerging adjunct to PFMT that can be worn during running to potentially assist symptom reduction. These garments are designed to lift and compress the pelvic floor (15–17) and support the lumbopelvic region (18), but they have received limited research attention to date. Possible discipline-focused theories regarding the effects of pelvic compression garments include alterations to running biomechanics, changes in perception, and enhanced pelvic floor support. The first theory proposes that pelvic compression garments affect hip and pelvis motion and thus the ability to attenuate pelvic shock. Because hip and pelvis motion likely contributes to the ability of a runner to attenuate shock, it is important to understand whether running biomechanics in postpartum women with PFD are altered when wearing pelvic compression garments.

A second theory is that pelvic compression garments affect individual perception (19). Specifically, it is unknown whether, or to what extent, these garments influence the perceived support provided by their compressive design, and how this may impact fear of experiencing PFD symptoms. Therefore, exploring whether pelvic compression garments alter the perception of support of a postpartum runner to the pelvic floor and surrounding region, and whether they influence PFD symptoms, is an important step towards understanding their biopsychosocial effects.

The third theory regarding the effects of pelvic compression garments relates to their interaction with levator hiatus distensibility (the capacity of the pelvic floor opening to expand). Wearing a pelvic compression garment daily for 12 weeks has been shown to reduce SUI among symptomatic women, an effect attributed to an increase in bladder neck height both at rest and during strain (16, 17). Preliminary research suggests that, in postpartum women who delivered vaginally, wearing a pelvic compression garment is more likely to result in an immediate lift in bladder neck height in those with greater levator hiatus distensibility compared with those with lower distensibility (19). While these studies provide some insights into the way in which pelvic compression garments might function to support the pelvic floor, they investigated their effects during static measurements or daily activity (16, 17, 19). Therefore, it remains unknown how these findings translate to pelvic floor support and symptom experience during higher-impact activities, such as running, or whether levator hiatus distensibility influences any biomechanical or perceptual interactions.

### Study aim

The primary aim of this study was to use a biopsychosocial approach to investigate the effect of a pelvic compression garment designed to lift and compress the pelvic floor on running biomechanics, perceived pelvic support, and perceived symptoms in postpartum runners with PFD. A secondary aim was to explore whether levator hiatus distensibility contributes to how postpartum women biomechanically respond to a compression garment during running and how it influences their perceptions.

## Materials and methods

### Study design

This exploratory study employed a randomised, repeated-measures crossover design in which test conditions were randomised and each participant served as their own within-person experiment and control condition. The CONSORT extension for pilot and feasibility trials was used to guide study conduct and reporting (20). During a single 2-h laboratory visit to Cardiff Metropolitan University, Cardiff, participants completed a pelvic floor assessment to measure PFM strength, endurance, and connective tissue support, followed by a treadmill running protocol to evaluate running biomechanics under two randomised conditions—in their own running shorts (control) and in a pelvic compression garment (experimental). The test order was randomised based on a unique participant number using an online list randomiser (https://www.random.org/lists/). Refer to Supplementary Appendix SI for an overview of participant flow through the study.

### Participants

Thirteen postpartum runners with self-reported symptoms of PFD were recruited via social media, research contacts, pelvic health physiotherapists, and parent and mother groups. A full overview of participant characteristics is outlined in Table 1. Self-reported PFD was quantified using the validated Australian Pelvic Floor Questionnaire (21, 22). Participants were required to have given birth within the last 5 years, be engaging in at least 150 min of moderate-intensity activity per week, have completed at least one 30-min run since giving birth [as data collection for this study was conducted concurrently with data collected for another study (8)], and be familiar with treadmill running. Participants were excluded if they were less than 12 weeks postpartum, currently pregnant or actively trying to conceive, had cardiovascular pathology, had previous gynaecological surgery for SUI, or had any lower limb injury or neurological impairment that affected their gait. All participants completed a written informed consent process to enrol in the study and were informed that they could withdraw at any time. Ethical approval was obtained from Cardiff Metropolitan University Ethics Committee (protocol number: Sta-2875), and all participants provided written informed consent before participating.

**Table 1:** Participant characteristics (*n* = 13).

Participant	Mean ± SD
Age (years)	38 ± 4
Height (m)	1.68 ± 0.06
Body mass (kg)	68.5 ± 9
Number of running sessions per week prior to pregnancy	3 ± 1
Distance run per week prior to pregnancy (km)	10 ± 10
Week returned to running postpartum	25 ± 17
Australian Pelvic Floor Dysfunction Score (*n*/40)	8 ± 3
Delivery mode for all births	n (%) or *median (range)*
Total number of births	30
Median number of births (range)	2 (1–4)
Vaginal	24 (80%)
Vaginal assisted	4 (13%)
Caesarean	2 (7%)
Episiotomy	9 (69%)
Obstetric anal sphincter injury	2 (15%)
Biomechanical descriptives	Mean ± SD
Self-selected running speed (m/s)	2.40 ± 0.20
Right anterior–posterior breast displacement (mm)^a^	16.43 ± 5.73
Right medial–lateral breast displacement (mm)^a^	23.20 ± 13.44
Right vertical breast displacement (mm)^a^	9.39 ± 3.41
Left anterior–posterior breast displacement (mm)^a^	11.91 ± 3.95
Left medial–lateral breast displacement (mm)^a^	17.55 ± 9.04
Left vertical breast displacement (mm)^a^	20.68 ± 11.85

a Breast motion was normalised to each participant's gait cycle. Displacement in each plane was calculated as the minimum–maximum distance between the origin of the trunk segment and each nipple marker across the entire gait cycle (70).

### Pelvic floor function and support

All pelvic floor assessments were carried out by an experienced pelvic health physiotherapist (GD) in a private room, with a senior researcher (IM) present to act as an intimate examination chaperone. PFM strength and endurance of each participant were assessed in crook lying and standing positions via digital vaginal palpation in accordance with the Modified Oxford Manual Muscle Testing Scale (23). The degree of participant pelvic organ and connective tissue support was assessed using the simplified POP-Q proforma (24–27). This included Gh + Pb measure at rest, during Valsalva, as well as the associated change score from rest to Valsalva, to quantify levator hiatus distensibility within the participant group. Since the modified POP-Q can only be performed in crook lying, pelvic organ and connective tissue support was also quantified using the Baden–Walker scale (28) in both lying and standing positions. Relevant testing notes were also recorded, such as whether the participant leaked urine or held back from executing a test cue.

### Compression garment intervention

**Figure 1:**
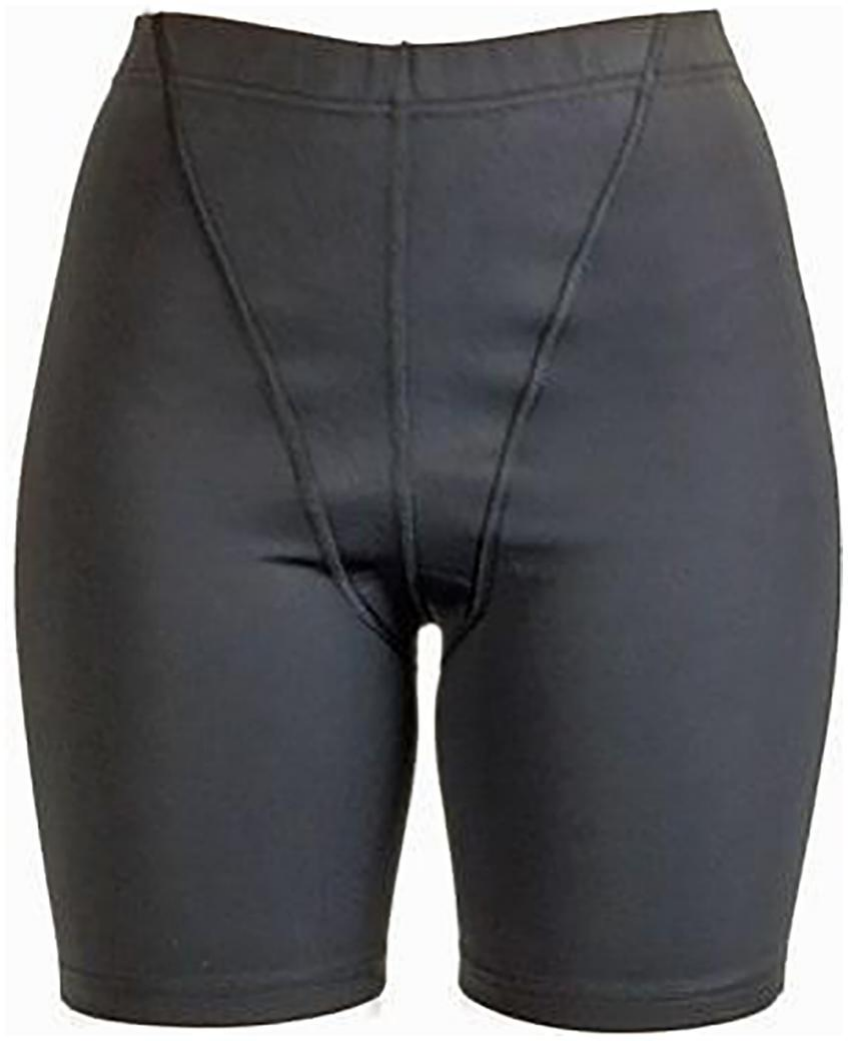
EVB Sport and Core pelvic compression garment. Permission to use image granted by EVB Sport and Core.

Each participant was individually sized for a pair of pelvic compression garment shorts (EVB Sport and Core, EVB Sport, Ireland; see Figure 1) prior to attending the laboratory for data collection. The sizing procedure involved using self-measurements (waist and hip circumference) and a single video call with the designer of the pelvic compression garment shorts (EB) to discuss and visualise the fit. This one-off input from the designer ensured that the best and intended fit of the pelvic compression garment was tested during the trial.

### Treadmill running protocol

**Figure 2:**
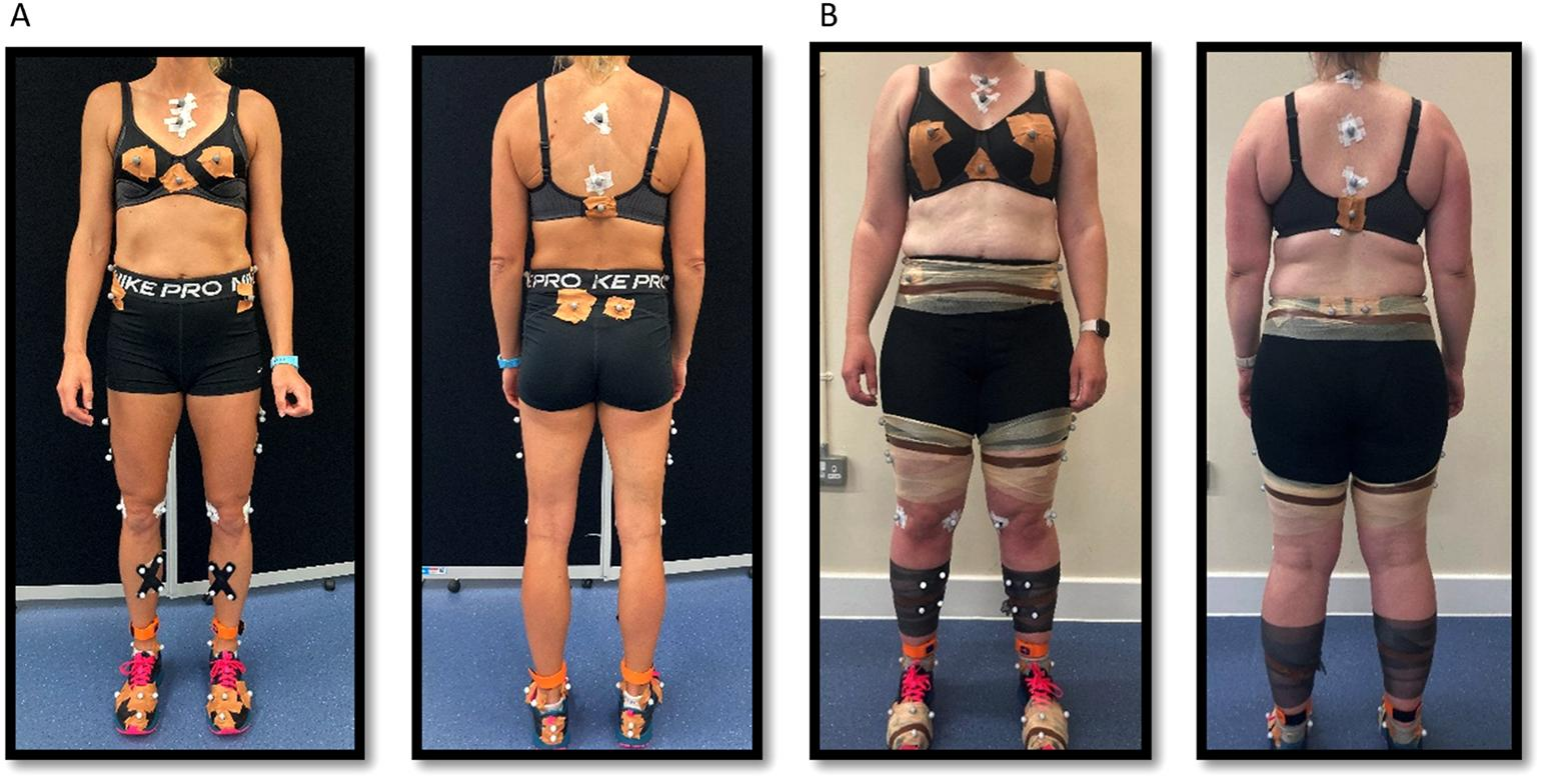
Participant examples showing how markers were attached to the thorax, pelvis, and lower limb, as per the University of Western Australia lower body, and the University of Portsmouth trunk and breast marker set. Participant **(A)** showing the marker placement during testing in her own shorts. Participant **(B)** showing the marker placement during testing while wearing the pelvic compression garment shorts. Markers were secured into position using underwrap tape. Inertial measurement units (IMU) are shown on the tibias in both participants. The pelvis IMU is not visible because it was placed directly onto the skin at the sacrum, between the left and right posterior superior iliac spines.

Following the PFM assessment, participants completed a treadmill running protocol in a biomechanics laboratory. Prior to dynamic data collection, fifty-one 14-mm retro-reflective markers were affixed to the thorax, breasts, pelvis, and lower limbs (Figure 2) as defined by the University of Western Australia lower body and thorax marker set (29). Static and functional calibration trials were captured to define body segments and joints using previously established and validated methods (29). Two inertial measurement units (IMUs; 225 Hz; Blue Trident, Vicon Motion Systems Ltd., Oxford, UK) were attached anteromedially over the distal tibia on each lower limb (Figure 2), following the methods previously described by Coltman et al. (8). In addition, an IMU was placed on the sacrum between the left and right posterior superior iliac spines (30). To ensure consistency throughout data collection, the same researcher (CC) secured all reflective markers and IMUs to the skin and shoes using tape.

Participants completed a 6-min familiarisation warm-up at an initial self-selected pace on the treadmill, followed by two 7-min running trials at the same self-selected running velocity. A 7-min trial duration was selected to ensure biomechanical parameters over 10 gait cycles reached a steady state and minimised variability (31, 32). Self-selected pace was determined by participants who were instructed to “run at a speed that you can comfortably run at for 30-min.” The order of intervention and control condition was randomised, and a rest period (∼5 min) was implemented between conditions. When the participant wore the experimental pelvic compression garment during the first trial, a longer rest period of 10 min was chosen to implement a washout period (19, 33, 34) and limit potential carryover effects of compression to the next trial. During minutes 5 and 6 of each 7-min trial, coordinate and analogue data were synchronously captured using a 15-camera three-dimensional motion analysis system (Vantage, Vicon, Oxford Metrics, UK, 250 Hz) and two force plates (Kistler Instruments AG, Winterthur, Switzerland, 1,000 Hz) embedded beneath a treadmill (Sprintex, Mar Systems Ltd., Ascot, UK).

### Biomechanical data collection and processing

A detailed overview of procedures used in this study can be found in an earlier publication in this series (8). In brief, biomechanical data analysis involved reconstructing and labelling marker trajectories using Vicon Nexus software (Oxford Metrics Ltd., Oxford, UK). Coordinate and analogue data were subsequently filtered and processed using Visual 3D (Visual3D x64 Student Edition v2024.08.5). Functional joint centre calculations were performed according to the methods described by Schwartz and Rozumalski (35) and outlined by HAS-Motion (36). A 50-Hz notch filter was applied to the analogue data, followed by a low-pass second-order Butterworth filter with a 12 Hz cut-off frequency, determined via residual analysis and visual inspection. Coordinate data underwent the same low-pass filtering with a cut-off frequency of 14 Hz, determined in the same manner as the analogue cut-off (37). Touchdown and toe-off events were identified from the vertical force data for 20 consecutive steps when the vertical force exceeded or dropped below a threshold of 80 N, respectively. Visual insights into the lab set-up and the collection and processing of Vicon and Visual 3D data are provided in Supplementary Appendix SII.

A customised MATLAB script (MATLAB, MathWorks Inc., MA, USA) aligned acceleration data to the global axes and corrected vertical acceleration to gravity (9.81 m.s^−1^) (38); then, a low-pass fourth-order Butterworth filter with a 10 Hz cut-off frequency, determined via residual analysis and visual inspection, was applied. Peak vertical acceleration of the pelvic IMU was identified for each step during stance. Consistent with Gruber et al. (39), frequency-domain shock attenuation (dB) was calculated, with positive values indicating a gain and negative values indicating signal attenuation. The integral of the transfer function within low (2–9 Hz) and high (9–22 Hz) frequency ranges was used to quantify the magnitude of shock attenuation. The area under the peak pelvic acceleration (g·s) curve was calculated using the area under the pelvic acceleration curves during each lower limb stance phase (40). Vertical instantaneous jerk represented the maximum time derivative of the acceleration curve during the stance phase.

All force magnitudes were normalised to body weight. When impact peaks were present, the magnitude of the impact peak (BW) was quantified, along with instantaneous and average loading rates (BW·s^−1^), which were calculated as the maximum time derivative of the vertical force during the middle 60% of the time between touchdown and impact peak and the magnitude of the impact peak divided by the time to impact peak, respectively. The active peak (BW) was defined as the maximum vertical ground reaction force during each midstance phase (40%–60%). Vertical impulse (BW·s) was calculated as the integral of the vertical force during the stance phase with respect to time. Change in leg length was determined from touch down to active peak, and leg stiffness (BW·m^−1^) was calculated as the active peak divided by the change in leg length (m).

Spatiotemporal variables were calculated according to the methods described by Coltman et al. (8) and included stride time (s), stance (ground contact) time (s), duty factor (%), stride frequency (Hz), and stride length (m). Pelvic oscillation across the gait cycle was defined as the displacement (maximum–minimum) of the centre of mass in the vertical plane. The mean of 20 steps was used to calculate the pelvic vertical oscillation. Left-sided trunk-to-pelvis (sagittal plane), hip adduction (frontal plane), and knee flexion (sagittal plane) angles were calculated relative to the laboratory coordinate system, with positive values denoting joint flexion (trunk-to-pelvis, knee) and adduction (hip). Frontal, sagittal, and transverse plane trunk and pelvis segment angles were also calculated, with positive values representing segment obliquity, flexion/tilt, and internal rotation, respectively. Angle data were normalised to 101 data points during the stance phase and averaged across 10 gait cycles.

### Perceived pelvic floor symptoms and garment support

Following each running trial, participants were asked a modified version of the symptom component questions of the PFD-SENTINEL (41). The PFD-SENTINEL is a screening tool used by sports medicine clinicians to identify the presence of PFD symptoms in female athletes. It provides a quick method to detect PFD symptoms related to a given activity. Modification of the PFD-SENTINEL tool involved asking the questions in the past, rather than the present, tense and contextualising them to the treadmill run. Participants were also asked questions to explore the real-time perceptual impact of each test condition in relation to perceived pelvic floor support, core support, and fear of experiencing PFD symptoms using a numerical rating scale (NRS) (0–10) with an interspersed visual guide to clarify the directionality of the score. The NRS is a widely used tool for assessing symptom intensity (typically pain) in clinical and research settings. The post-run test questions and associated NRS are provided in the Supplementary Appendix SIII.

### Statistical analysis

The means of the left and right sides for all biomechanical variables were calculated. Combined left–right mean differences in biomechanical variables over 20 steps were analysed using descriptive statistics (mean and standard deviation). To determine whether a pelvic compression garment affects within-person running biomechanics or perception, paired-sample *t*-tests were conducted on all dependent variables (42). Linear regression models were then used to explore levator hiatus distensibility as a potential predictor of the biomechanical or perceptual effect of a pelvic compression garment (43). Specifically, separate stepwise linear regression models were fit for each variable of interest, as this technique utilises an automatic procedure to determine the most appropriate predictor variables and evaluate their order of importance (44). It is considered valuable when theoretical arguments and expert opinion are used to select the initial list of predictors (45), as is the case in this exploratory study. In each model, the dependent variable was the change score between the experimental pelvic compression garment and control conditions. The independent variables were Gh + Pb measurements (Gh + Pb maximum length on Valsalva and Gh + Pb change length from rest to Valsalva) and the mean-centred biomechanical or perceptual value without a pelvic compression garment. Mean-centred covariates and change scores for the matched tests were chosen for the regressions because they are understood to detect more sensitive differences (46).

A bespoke MATLAB script was used for statistical parametric mapping (SPM) (47) of the continuous biomechanical data to determine whether there were differences in kinematic variables across the stance phase of the gait cycle between conditions. During SPM analysis, the mean of the left and right kinematic variables was assessed separately across frontal, sagittal, and transverse planes, and analyses were performed on the left lower limb and segment data.

Homoscedasticity and normality of the data were assessed via visual inspection of test variable residuals for the regression models. All statistical analyses were performed in SPSS [IBM SPSS Statistics version 28.0.0.0 (190)], Excel [Microsoft Excel for Microsoft 365 MSO (version 2410, Build 16.0.18129.20158) 64-bit], or MATLAB (MATLAB, MathWorks Inc., MA, USA), and alpha was set at 0.05.

## Results

**Figure 3:**
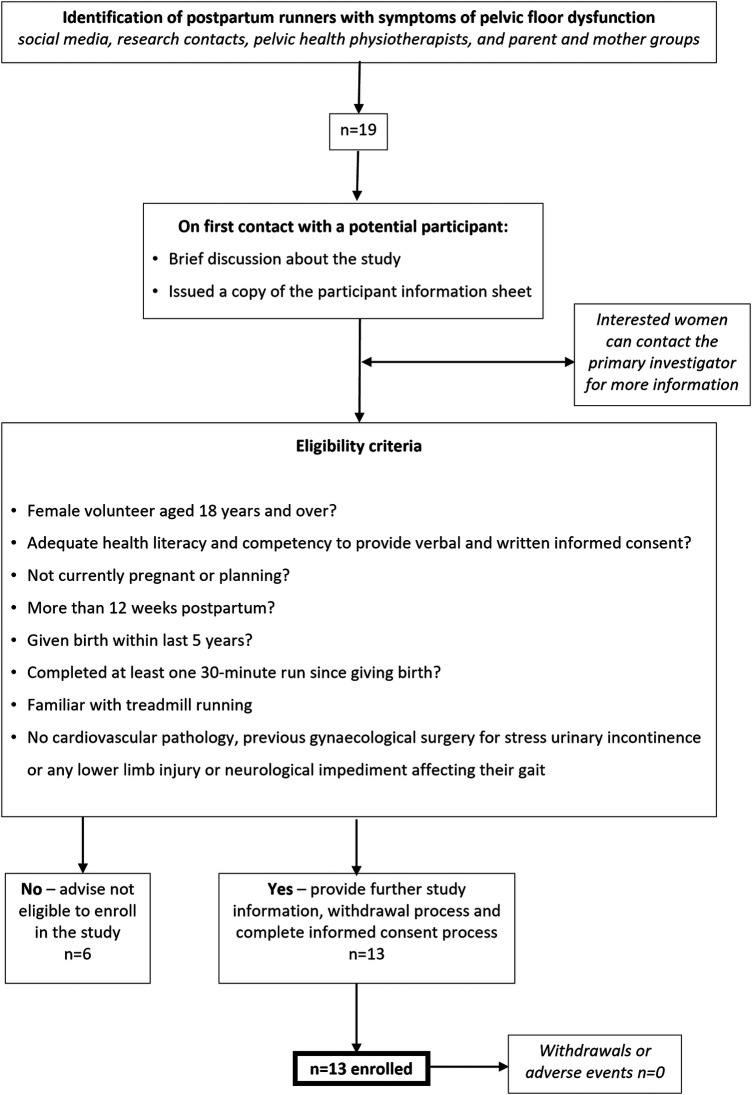
Participant recruitment process.

Thirteen participants enrolled in and completed the study with no adverse events. Figure 3 provides an overview of the participant recruitment process. All data were normally distributed. Example Q–Q plots and scatterplots used for visual inspection during statistical analyses are given in Supplementary Appendix SIV. The mean (SD) is also reported for all variables to describe the data distribution.

### Pelvic floor function and support

The PFM strength and endurance data are shown in Table 2. Measures of PFM strength and endurance were similar across the two assessment positions (crook lying vs. standing), with a mean strength grade of 3 (out of 5) and sustained hold of 8 s according to the Modified Oxford Manual Muscle Test (23). Pelvic floor connective tissue support and levator hiatus distensibility of the participants are also reported in Table 2.

**Table 2:** Baseline participant pelvic floor data.

Pelvic floor baseline	Mean ± SD
Modified Oxford Manual Muscle Testing	
Crook lying: pelvic floor muscle strength (/5)	3 ± 1
Number of fast repetitions (/10)	9 ± 1
Number of sustained contractions (/10)	4 ± 2
Length of time for sustained contractions (s)	8 ± 3
Standing: Pelvic floor muscle strength (/5)	3 ± 1
Number of fast repetitions (/10)	9 ± 2
Number of sustained contractions (/10)	4 ± 3
Length of time for sustained contractions (s)	8 ± 3
POP-Q	
Total score (/4)	1 ± 1
Gh + Pb at rest (cm)	6 ± 1
Gh + Pb Valsalva, maximum measure of 3× Valsalva (cm)	7 ± 1
Gh + Pb change score (rest to Valsalva (cm)	1 ± 1
Leak during Valsalva (*n*; %)	2 (15%)
Withhold during Valsalva (*n*; %)	3 (23%)
Bayden–Walker score (/3)	
Crook lying	1 ± 1
Standing	1 ± 0

### Biomechanical data

**Figure 4:**
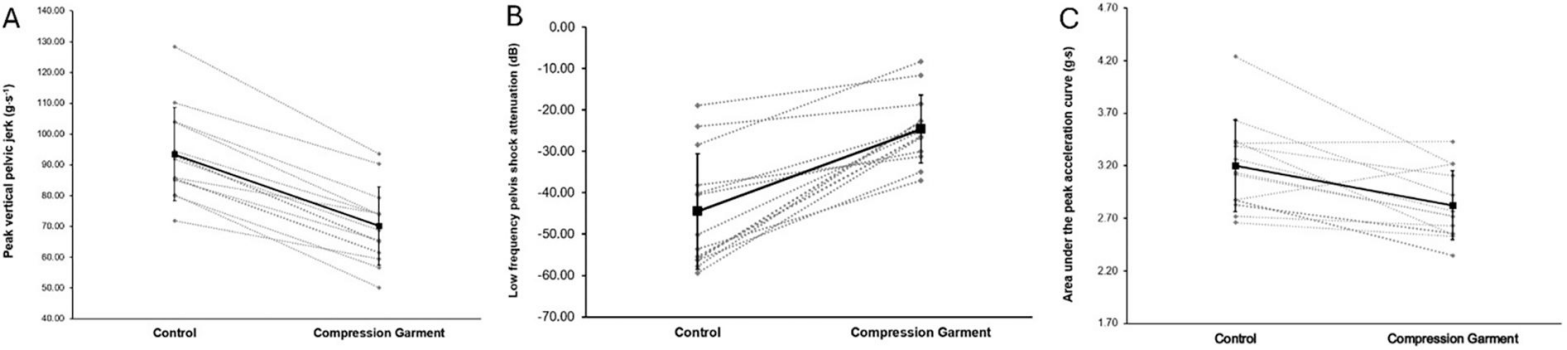
Select repeated-measures biomechanical variable outcomes for each participant (*n* = 13) for the control and experimental pelvic compression garment conditions. **(A)** Peak vertical pelvic jerk (g·s^−1^), **(B)** low-frequency pelvis shock attenuation (dB), and **(C)** area under the peak pelvic acceleration curve (g·s).

The mean and standard deviation for pelvic loading and shock attenuation, kinetic and spatiotemporal variables are reported for the control and experimental pelvic compression garment conditions in Table 3 and visually displayed for a selection of variables in Figure 4. Only pelvic loading and shock attenuation variables exhibited differences between conditions. Specifically, peak pelvic jerk, low-frequency pelvic shock attenuation, and area under the peak pelvic acceleration curve decreased in the experimental pelvic compression garment condition compared to the control condition.

**Table 3:** Running-related variables across several defined domains in the control and experimental compression garment conditions.

Domain	Variable^†^	Paired *t*-test			
		Control, mean ± SD	Compression garment, mean ± SD	*p*-Value	(*g*)
Accelerometer	Peak pelvic jerk (g·s^−1^)	93.38 ± 15.11	77.82 ± 13.31	**0.00**	1.40
	Freq shock attenuation (low) (dB)	−44.51 ± 13.91	−24.61 ± 8.25	**0.00**	1.69
	Freq shock attenuation (high) (dB)	−2.26 ± 38.61	−22.05 ± 28.84	0.07	0.56
	Area under the peak pelvic acceleration curve (g·s)	3.20 ± 0.44	2.82 ± 0.33	**0.00**	0.94
Kinetic	Average loading rate (BW·s^−1^)	37.32 ± 6.36	37.21 ± 10.05	0.10	0.01
	Instantaneous loading rate (BW·s^−1^)	54.37 ± 9.18	54.94 ± 8.54	0.09	0.06
	Leg stiffness (BW·m^−1^)	35.59 ± 10.83	40.28 ± 11.48	0.12	0.41
	Vertical impulse (*N*)	65.42 ± 17.72	65.09 ± 16.07	0.89	0.02
	Vertical impulse (BW·s)	0.10 ± 0.03	0.10 ± 0.03	0.82	0.02
	Vertical active peak (BW)	2.21 ± 0.25	2.16 ± 0.21	0.41	0.19
Spatiotemporal	Stride length (m)^a^	1.76 ± 0.20	1.75 ± 0.25	0.84	0.05
	Stride frequency (Hz)^b^	1.36 ± 0.07	1.36 ± 0.08	0.96	0.01
	Steps per minute	163.08 ± 8.06	163.19 ± 9.61	0.96	0.01
	Ground contact time (s)^c^	0.27 ± 0.02	0.27 ± 0.02	0.10	0.13
	Vertical COM displacement (m)	0.09 ± 0.02	0.09 ± 0.01	0.40	−0.01
	Duty factor (%)	36.12 ± 3.49	36.91 ± 4.28	0.42	0.19
Perceptual	Pelvic floor support (/10)	2 ± 2	7 ± 2	**0.00**	2.64
	Core support (/10)	4 ± 3	8 ± 1	**0.00**	2.12
	Fear of PFD symptoms (/10)	6 ± 3	4 ± 3	**0.01**	0.87
	PFD-SENTINEL (/5)	1 ± 1	1 ± 1	0.75	0.10

Bold values indicate the pre-determined significance threshold of <0.05 has been reached.

(*g*) unbiased estimate of effect size calculated using Hedges *g* correction.

a Stride length was calculated as a product of stride time and running speed (m.s^−1^).

b Stride frequency (Hz) was calculated as the inverse of stride time.

c Ground contact time was defined as the time between initial foot contact and toe-off for the same foot.

† Biomechanical variable data were collected in the vertical plane during stance.

Stepwise linear regression models were then fit for each outcome variable that demonstrated a significant difference between conditions in the paired *t*-tests or was considered to be of clinical or scientific interest by the research team (Table 4). For all biomechanical variable models, neither measure of levator hiatus distensibility was retained in the stepwise selection procedure. Instead, the final models included an intercept, representing the change in the variable between conditions (control vs. experimental pelvic compression garment) while adjusting for the control condition value, and an effect of the control value. Although Gh + Pb values were excluded from all biomechanical variable models, their individual coefficients are reported in Table 4 for transparency, alongside the intercept values included in the final models. Residual plots confirmed adequate model fit.

**Table 4:** Stepwise linear regression using the mean-centred control score, Gh + Pb maximum length, and Gh + Pb change score as independent variables and the matched change score between conditions (control and experimental pelvic compression garment conditions) as the dependent variable.

	Stepwise linear regression										
	Final model					Excluded from the model					
Variable^a^	Independent variable	*β*	SE	*p*-Value	*R* ^2^_adj_	Independent variable	β*	*t*-Value	*p*-Value	PC	CST
Peak pelvic jerk (g·s^−1^)	Intercept	−23.23	1.60	**<0**.**00**	0.26	Max Gh + Pb	−0.19	−0.68	0.51	0.21	0.83
	Mean-centred value without CGs	−0.25	0.11	**0**.**04**		Change Gh + Pb	0.04	−0.14	0.89	−0.04	0.98
Freq. shock attenuation (low) (dB)	Intercept	19.90	1.82	**<0**.**00**	0.62	Max Gh + Pb	−0.06	−0.29	0.78	−0.09	0.92
	Mean-centred value without CGs	−0.62	0.14	**<0**.**00**		Change Gh + Pb	0.04	−0.23	0.82	−0.07	0.99
Freq. shock attenuation (high) (dB)	Intercept	−3.57	0.40	**<0**.**00**	0.63	Max Gh + Pb	0.02	−0.13	0.90	−0.04	0.97
	Mean-centred value without CGs	−0.84	0.18	**<0**.**00**		Change Gh + Pb	−0.09	−0.43	0.68	−0.14	0.81
Average loading rate (BW·s^−1^)	Intercept	−2.34	3.65	0.53	0.58	Max Gh + Pb	−0.24	−1.01	0.31	−0.38	0.92
	Mean-centred value without CGs	−2.11	0.57	**0**.**01**		Change Gh + Pb	−0.17	−0.75	0.48	−0.27	0.96
Area under the peak pelvic acceleration curve (g·s)	Intercept	−0.37	0.08	**<0**.**00**	0.41	Max Gh + Pb	−0.05	−0.21	0.84	−0.07	0.84
	Mean-centred value without CGs	−0.58	0.19	**<0**.**01**		Change Gh + Pb	−0.29	−1.33	0.21	0.39	1.00
Pelvic floor support (/10)	Intercept	4.85	0.56	**<0**.**00**	0.45	Max Gh + Pb	−0.05	−0.18	0.81	−0.57	0.81
	Mean-centred value without CGs	−1.20	0.37	**0**.**01**		Change Gh + Pb	−0.28	−1.24	0.87	−0.36	0.87
Core support (/10)	Intercept	4.34	0.38	**<0**.**00**	0.73	Max Gh + Pb	0.14	0.87	0.41	0.27	0.91
	Mean-centred value without CGs	−0.91	0.16	**<0**.**00**		Change Gh + Pb	−0.09	−0.55	0.59	−0.17	1.00
PFD-SENTINEL (/5)	Intercept	−0.08	0.20	0.71	0.28	Max Gh + Pb	−0.14	−0.50	0.63	−0.16	0.80
	Mean-centred value without CGs	−0.65	0.28	**0**.**04**		Change Gh + Pb	−0.20	−0.73	0.49	−0.22	0.83
Fear of PFD symptoms (/10)*	Intercept	5.77	4.80	0.26	0.25	-	-	-	-	-	-
	Mean-centred value without CGs	−0.40	0.24	0.13		Change Gh + Pb	0.33	0.41	0.69	0.14	0.61
	Max Gh + Pb	−1.21	0.71	0.12		-	-	-	-	-	-

*β*, standardised beta coefficient; SE, standard error; *R*^2^_adj_, adjusted *R*-squared; *p*-value set at <0.05; β*, potential standardised beta coefficient if the variable was included in the model; *t*-value, standard errors of the estimated coefficients away from zero; PC, partial correlation; CST, collinearity statistics tolerance; CGs, pelvic compression garments; Max Gh + Pb, maximum genital hiatus plus perinal body length (cm) during Valsalva; Change Gh + Pb, change score in genital hiatus plus perineal body length (cm) between rest and Valsalva; *stepwise regression for variable run through R due to errors in SPSS.

a Biomechanical variable data were collected in the vertical plane during stance.

Bold values indicate the pre-determined significance threshold of <0.05 has been reached.

**Figure 5:**
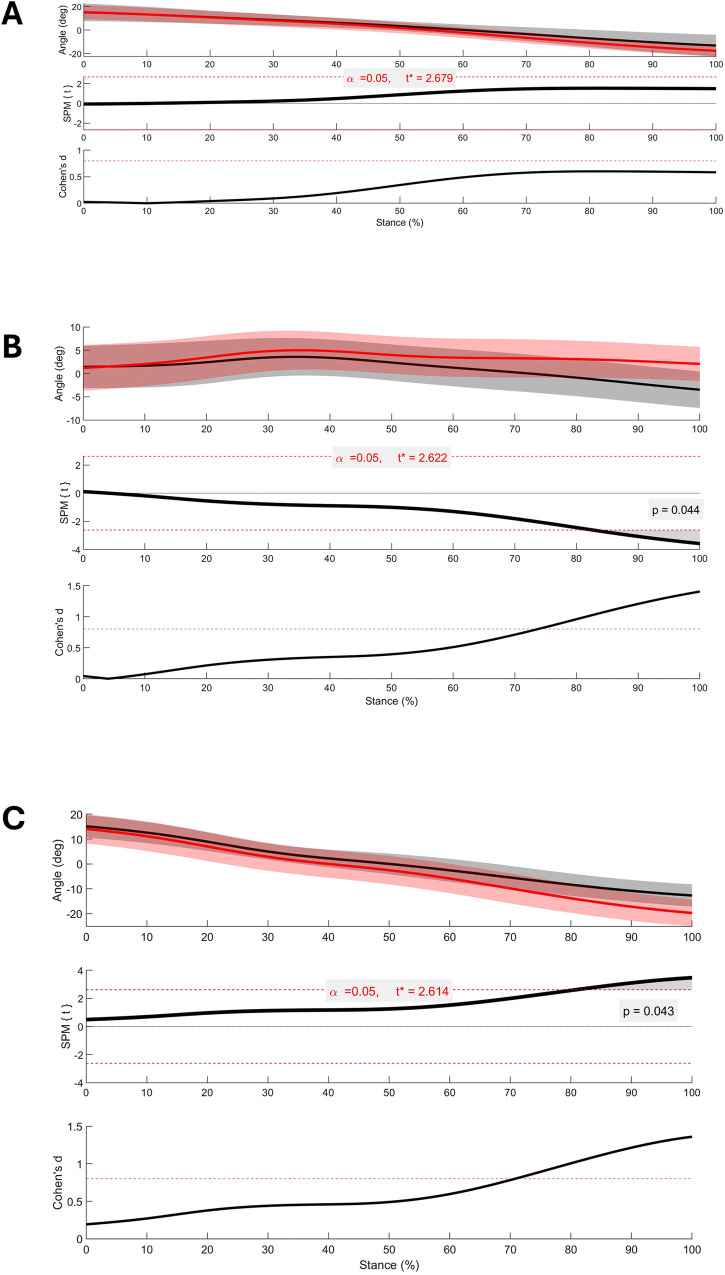
Statistical parametric mapping (SPM) of global trunk **(A)**, global pelvis **(B)**, and trunk-to-pelvis **(C)** angles during the stance phase of gait according to the left lower limb. Angle plots: solid black line—control condition; solid red line—experimental pelvic compression garment condition; solid lines represent group means; shaded regions around the mean are +/-1 SD. SPM plots: solid black line represents SPM comparison for corresponding angle plot with significance threshold set at alpha = 0.05 (red dashed line) and grey shaded areas representing timepoints reaching significance. Cohen's *d* plots: the solid black line represents effect size, which was computed using Cohen's *d* at each timepoint. The dashed red line represents a large effect size (0.8).

The SPM analysis identified significant differences with large effects between conditions for two left-sided pelvic angles between 80% and 100% of stance in the transverse plane. Participants exhibited reduced left-sided internal-to-external pelvic rotation motion (*p* = 0.04) in the experimental pelvic compression garment condition, retaining the pelvis in a neutral position at left push-off, compared to the control condition, which continued into external pelvic rotation. In addition, there was greater separation between trunk and pelvis segments, resulting in a larger axial trunk-to-pelvis joint rotation angle (*p* = 0.04), due to the experimental pelvic compression garment condition holding the pelvis in a neutral position during the propulsive component of stance (Figure 5). No significant differences were identified between conditions for any other pelvic or trunk angles using SPM.

### Perceived pelvic floor support and symptom variables

**Figure 6:**
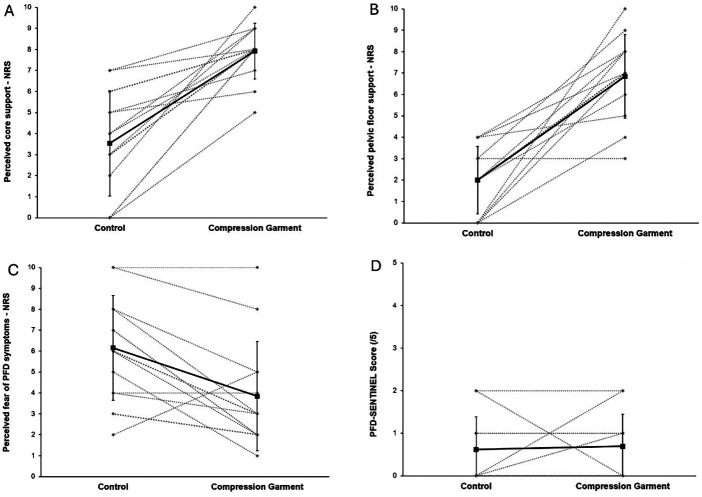
Select repeated-measures perceptual variable outcomes for each participant (*n* = 13) in the control and experimental compression garment conditions for perceived core support (**A**), perceived pelvic floor support (**B**), perceived fear of PFD symptoms (**C**), and PFD-SENTINEL score (**D**).

Mean and standard deviation data for perceptual variables and symptom scores in the two conditions are presented in Table 3 and visually depicted in Figure 6. A significant effect of condition was identified for perception of pelvic floor support (*p* ≤ 0.00), core support (*p* ≤ 0.00), and fear of experiencing pelvic floor symptoms (*p* = 0.01). No effect of condition was detected for the modified PFD-SENTINEL symptom score (*p* = 0.75).

When perceptual variables were entered into a stepwise linear regression model, significant changes were detected between the experimental pelvic compression garment condition for pelvic floor support, core support, and PFD-SENTINEL symptom score, when controlling for the mean-centred control value (Table 4). The addition of Gh + Pb to the stepwise regression only improved the model output for *fear of PFD symptoms*; however, it did not reach statistical significance (*p* = 0.12). Gh + Pb independent variables were excluded from variable models for perceived pelvic floor support, perceived core support, and the PFD-SENTINEL symptom score.

## Discussion

This study explored the biopsychosocial impact of a pelvic compression garment targeting the pelvic floor in postpartum runners. Rather than focusing on a single domain, our study explored multiple interrelated domains in accordance with the biopsychosocial model (48, 49): running biomechanics (bio), perceived garment support and symptoms (psychosocial), and whether levator hiatus distensibility predicts these effects (biopsycho). Our findings identify relevant biomechanical and perceptual effects of pelvic compression garments and offer insight into their potential role in supporting postpartum runners with PFD. Specifically, we identified decreased peak pelvic jerk, low-frequency pelvic shock attenuation, area under the peak pelvic acceleration curve, and transverse plane pelvic rotation when postpartum women ran in a pelvic compression garment compared to the control condition. In addition, findings demonstrated decreased fear of experiencing pelvic floor symptoms and improved perceptions of core and pelvic floor support in the pelvic compression garment condition compared to the control condition. No significant interactions were identified for Gh + Pb measures for any biomechanical or perceptual variable, and the addition of pelvic compression garments did not significantly affect actual symptom experience during running. Insights into each of these findings are discussed further below.

### Do pelvic compression garments alter running biomechanics in symptomatic postpartum runners?

Peak pelvic jerk, low-frequency pelvic shock attenuation, and area under the peak pelvic acceleration curve were reduced when participants wore a pelvic compression garment. In other words, when postpartum women ran in a pelvic compression garment, they exhibited a smoother pelvic running motion by reducing the rate of change in pelvic acceleration and attenuated shock through less active mechanisms (e.g., segment orientation, eccentric muscle actions, and joint stiffness). It is theorised that pathology modulates gait smoothness, with jerkier movement occurring in the presence of pathology, a finding supported by our previous work on PFD in postpartum runners (8). This current study builds on this theory, as the findings indicate that pelvic compression garments may offer external support that facilitates the production of a smoother running gait in postpartum women experiencing pathology (i.e., PFD). In addition, the pelvic compression garment appears to assist shock attenuation at the pelvis by potentially offloading the pelvic floor and lumbopelvic muscles and tissues. Lowering the active requirements needed to attenuate shock at the pelvis is relevant to female runners, as previous research highlights that runners with SUI are exposed to higher shock attenuation demands at the pelvis compared to continent runners (9). In loaded single-limb activities such as running, the stance hip must control the femur and pelvis in response to higher-impact forces while also supporting the mass of the head, upper limbs, trunk, and non-stance leg (50). Such dynamic stability of high-velocity lumbopelvic rotation during running is understood to be achieved through the function of the deep hip rotators and gluteal muscles (50, 51). Thus, wearing a pelvic compression garment may support the body's ability to passively dampen pelvic oscillations, in a similar manner to how lower limb compression garments act on the thigh and calf muscles (52). In postpartum women, such support from a pelvic compression garment may be particularly beneficial, as pregnancy and vaginal childbirth can impact the capacity of the pelvic floor tissues to dampen pelvic floor oscillations (53). Collectively, these novel findings indicate that mechanical changes to running gait can occur in postpartum women with PFD with the use of a pelvic compression garment. The mechanical changes likely help to offload the surrounding muscles and tissues and improve the loading capacity of the pelvic floor (54–56).

Interestingly, while most biomechanical research focuses on the impact phase of stance, where pelvic floor symptoms are likely to be provoked, our findings demonstrate novel insights into the kinematics of the propulsive phase of stance. Compared to the control condition, wearing a pelvic compression garment resulted in greater transverse (axial) right rotation in the trunk-to-pelvis angle, driven by a reduction in the transverse left (external rotation) pelvis segment angle towards the end of stance (80%–100%). No differences in lower limb kinematics were observed. The kinematic waveforms of the trunk and pelvis segment angles in the pelvic compression garments are similar to those reported by Schache and colleagues (57) in their work on lumbar spine and pelvis angles during running. In contrast, the control condition unexpectedly placed the pelvis in transverse left (external) rotation towards the end of stance, resulting in a greater pelvic range of motion. Minimising the relative motion between the trunk and pelvis has been observed in runners with low back pain (58) and in postpartum women (10, 59), possibly serving as a neural strategy to reduce pain or to increase lumbopelvic control to overcome perinatal structural changes. In terms of PFD, it is conceivable that the timing of PFM activation may be disrupted when in-phase trunk and pelvis, rather than trunk-only, coordination occurs at push-off. The PFMs act in an anticipatory and feedforward manner (60, 61), and the timing of their activation in relation to the trunk muscles is likely relevant for maintaining continence (62). If changes in trunk muscle activation occur due to coordination coupling of the trunk and pelvis when lumbopelvic stability is compromised, this may reduce the effectiveness of the force closure mechanism at the pelvis. Pelvic force closure refers to altered joint reaction forces, created by taut muscles and connective tissues, that create a perpendicular compressive reaction force at the pelvis in response to the gravitational and ground reaction forces (63). Our data indicate that wearing a pelvic compression garment restricts transverse pelvic motion in postpartum women with PFD, promoting a trunk–pelvis coordination pattern similar to that of healthy runners, which may improve the effectiveness of pelvic force closure. Further research is needed to substantiate this.

### Do pelvic compression garments alter the perceptions of postpartum runners?

A significant effect of condition was identified for several perceptual variables (Table 3). Perceived core and pelvic floor support significantly improved by 4 and 5 points, respectively, on a 0–10 NRS comparing the pelvic compression garment condition to the control condition. Interestingly, while the perceived fear of experiencing symptoms significantly decreased by a mean of 2/10 on the NRS, an improvement in self-reported symptom experience after the laboratory test run was not observed in our study, according to the modified PFD-SENTINEL score. This may suggest that the PFD-SENTINEL tool is not sensitive enough to capture smaller changes of 2 units on the NRS, that perceptual changes may not correspond to immediate symptom reduction, or that there is a difference in perceived fear of symptoms and actual symptom experience.

Trying to facilitate improvements in perceived PFD and kinesiophobia is considered relevant for postpartum runners, as pelvic floor symptoms and fear of movement have been identified as key barriers to postpartum women returning to running (5). As such, pelvic compression garments could offer a low-cost and ecological solution.

### Does levator hiatus distensibility in postpartum runners influence the biomechanical or perceptual effect of pelvic compression garments?

This is the first study to explore whether levator hiatus distensibility interacts with running biomechanics or perception. In our symptomatic postpartum women, no models were significantly improved by the addition of levator hiatus distensibility as an independent variable. This suggests that levator hiatus distensibility does not appear to influence how postpartum women with PFD biomechanically or perceptually respond to pelvic compression garments and therefore offers limited predictive value. It is possible that this lack of a relationship is largely due to the small sample size in this study and that only certain variables had sufficient power to detect significance. Other substantive explanations could be that the level of compression provided by the pelvic compression garment (64) was not high enough to elicit a reduction in distensibility or that measuring distensibility in a crook-lying position is not the most functional approach when examining running biomechanics. We assessed PFM function and aspects of pelvic organ support (Bayden–Walker scale for pelvic organ descent) in both crook-lying and standing positions (Table 2) and observed no differences in the mean magnitudes for any measure. Although this was not possible for levator hiatus distensibility, these findings may indicate that a standing measure would elicit similar magnitudes to those obtained in crook lying. Conversely, our cohort had low symptom severity, and while half of the participants (*n* = 7) reached a clinical threshold of 7 cm Gh + Pb distensibility during the largest of three maximum Valsalva cues (65), only one participant exceeded this threshold (9 cm). This indicates that only half of our sample had clinically recognised levator hiatus distensibility and, for those who did, the clinical relevance was mild. One theory regarding the use of pelvic compression garments for pelvic floor support is that they may only provide an appropriate level of compression for individuals with moderate to severe clinical distensibility, specifically, those with Gh + Pb values exceeding 7 cm on Valsalva. This is tentatively supported by prior exploratory research demonstrating that women who delivered vaginally and had larger levator hiatus distensibility were more likely to exhibit an immediate increase in bladder neck height when wearing a pelvic compression garment (19).

Notably, while running is understood to increase the levator hiatus size and lower bladder neck height (66, 67), the relationship to SUI symptoms is more complex than levator hiatus size alone. For example, other factors such as genetic urethral predisposition (68, 69) and low energy availability (3) are also understood to contribute to SUI and may explain why half of our sample met the criteria for PFD yet did not demonstrate clinical levator hiatus distensibility. It is therefore likely that some degree of distensibility in the levator hiatus tissues is normal and can be accommodated without exposing or worsening PFD symptoms— at least until a clinical distensibility threshold is reached or additional risk factors interfere to expose symptoms.

Consequently, our data do not support the theory that levator hiatus distensibility interacts with how symptomatic postpartum women respond biomechanically or perceptually to pelvic compression garments. This highlights the need to further investigate pelvic compression garments that offer differing levels of compression targeted to the levator hiatus region (64), particularly in cohorts with higher PFD symptom expression and greater levator hiatus distensibility.

### Limitations

Given the exploratory nature of this study, no prior data were available to inform a sample size estimate. Sample size was determined based on practical considerations and resource availability. A *post-hoc* power calculation for the paired *t*-tests demonstrated that this study was adequately powered (>80%) to detect differences for dependent variables with a large effect size between conditions but was not adequately powered for dependent variables with a smaller hypothesised effect size. In addition, the variability of pelvic floor symptom domains (e.g., SUI, POP, pain) introduces potential confounders, and without sufficient sample size to carry out subgroup analyses, insights were limited. Moreover, while all participants self-reported symptoms, the overall severity of PFD was mild. It is possible that participants with more severe PFD symptoms may demonstrate a greater effect from the pelvic compression garment but may not have met our inclusion criteria of being able to run for 30 min or may have been reluctant to attend the laboratory due to fear or self-consciousness about displaying symptoms. However, a laboratory visit was necessary to undertake a comprehensive kinematic and kinetic biomechanical analysis for this study. Finally, although measures were taken to minimise unintentional bias regarding the pelvic compression garment, such as randomised test ordering, washout periods, and researcher neutrality, participants were aware when they were wearing a garment different from their own shorts. It is unknown to what extent a placebo effect may have influenced the perceptual data. Nevertheless, the significant findings in pelvic-related running biomechanical variables provide insight and rationale supporting the perceived differences.

### Clinical implications and future research directions

Pelvic compression garments may offer a low-cost option to support postpartum runners with PFD. Future research is needed to investigate varying levels of pelvic compression garments in cohorts with higher PFD symptom expression and greater levator hiatus distensibility. Research is also required to determine the clinical significance of reduced fear of movement and to investigate the relationship between perceived fear of symptoms and actual symptom experience, e.g., by utilising objective measures of SUI. Designing randomised controlled trials that compare pelvic compression garments with a sham garment is recommended. In addition, future studies could assess participants during their normal running training to mitigate some of the limitations associated with a laboratory testing design.

## Conclusion

Pelvic compression garments appear to alter biomechanics in postpartum women with symptoms of PFD in a way that produces a smoother running gait and restricts transverse pelvis motion, promoting trunk–pelvis coordination similar to that observed in healthy runners. Using a biopsychosocial approach alongside biomechanical alterations, it was also identified that wearing a pelvic compression garment increased perceived core and pelvic floor support and decreased fear of experiencing PFD symptoms compared to the control condition. Although biomechanical and perceptual changes were observed, levator hiatus distensibility did not appear to interact with how symptomatic postpartum women responded to wearing a pelvic compression garment. Future work exploring different levels of compression with greater clinical levator hiatus magnitudes and objective measures of pelvic floor symptoms in postpartum women would enhance our understanding of the use of pelvic compression garments as an adjunctive strategy for managing PFD.

## Data Availability

The raw data supporting the conclusions of this article will be made available by the authors, without undue reservation.

## References

[1] Bump RC Norton PA. Epidemiology and natural history of pelvic floor dysfunction. Obstet Gynecol Clin North Am. (1998) 25(4):723–46. 10.1016/S0889-8545(05)70039-5 9921553

[2] Delancey JO Kane Low L Miller JM Patel DA Tumbarello JA. Graphic integration of causal factors of pelvic floor disorders: an integrated life span model. Am J Obstet Gynecol. (2008) 199(6):610.e1–5. 10.1016/j.ajog.2008.04.001 18533115 PMC2764236

[3] Mountjoy M Ackerman KE Bailey DM Burke LM Constantini N Hackney AC 2023 International Olympic committee’s (IOC) consensus statement on relative energy deficiency in sport (REDs). Br J Sports Med. (2023) 57(17):1073–97. 10.1136/bjsports-2023-106994 Erratum in: Br J Sports Med. 2024 February 7;58(3):e4. doi: 10.1136/bjsports-2023-106994corr1. PMID: 37752011. 37752011

[4] Knol-de Vries GE Blanker MH. Prevalence of co-existing pelvic floor disorders: a scoping review in males and females. Continence. (2022) 2:100028. 10.1016/j.cont.2022.100028

[5] Moore IS James ML Brockwell E Perkins J Jones AL Donnelly GM. Multidisciplinary, biopsychosocial factors contributing to return to running and running related stress urinary incontinence in postpartum women. Br J Sports Med. (2021) 55(22):1286–92. 10.1136/bjsports-2021-104168 34144950

[6] James ML Moore IS Donnelly GM Brockwell E Perkins J Coltman CE. Running during pregnancy and postpartum, part A: why do women stop running during pregnancy and not return to running in the postpartum period? J Womens Health Phys Ther. (2022) 46(3):111–23. 10.1097/JWH.0000000000000228

[7] Donnelly GM James ML Coltman CE Brockwell E Perkins J Moore IS. Running during pregnancy and postpartum, part B: how does running-related advice and guidance received during pregnancy and postpartum affect women’s running habits? J Womens Health Phys Ther. (2022) 46(3):124–31. 10.1097/JWH.0000000000000240

[8] Coltman CE Donnelly GM von Lieres Und Wilkau H Moore IS. Is there an association between symptoms of pelvic floor dysfunction, running kinetics, and pelvic acceleration in postpartum women? J Appl Biomech. (2025) 41(3):258–70. 10.1123/jab.2024-0274 40204278

[9] Campos NC Fonseca ST Nunes LJ Ramos Rodrigues SI Figueiredo EM Araújo PA Inefficient impact absorption and reduced shock attenuation in female runners with stress urinary incontinence. J Biomech. (2025) 187:112753. 10.1016/j.jbiomech.2025.112753 40403602

[10] Provenzano SG Hafer JF Peacock JBS Kempner S Zendler JD Agresta CE. Restriction in pelvis and trunk motion in postpartum runners compared with Pre-pregnancy. J Womens Health Phys Ther. (2019) 43(3):119–26. 10.1097/JWH.0000000000000129

[11] Bagwell JJ Avila E Reynolds N Smith JA Valenzuela K Katsavelis D. Running biomechanics differ during and after pregnancy compared to females who have never been pregnant. Gait Posture. (2024) 109:277–83. 10.1016/j.gaitpost.2024.02.004 38377744

[12] NICE. Pelvic floor dysfunction: prevention and non-surgical management. National Institute of Clinical Excellence (2021). NG210. Available online at: https://www.nice.org.uk/guidance/ng210 (Accessed August 4, 2025). 35438877

[13] Dumoulin C Cacciari LP Hay-Smith EJC. Pelvic floor muscle training versus no treatment, or inactive control treatments, for urinary incontinence in women. Cochrane Database Syst Rev. (2018) 10(10):Cd005654. 10.1002/14651858.CD005654.pub4 30288727 PMC6516955

[14] Donnelly GM Moore IS. Sports medicine and the pelvic floor. Curr Sports Med Rep. (2023) 22(3):82–90. 10.1249/JSR.0000000000001045 36866951

[15] Szkwara JM Milne N Rathbone E. A prospective quasi-experimental controlled study evaluating the use of dynamic elastomeric fabric orthoses to manage common postpartum ailments during postnatal care. Women’s Health. (2020) 16:1745506520927196. 10.1177/1745506520927196 PMC7290251 32525761

[16] Okayama H Ninomiya S Naito K Endo Y Morikawa S. Effects of wearing supportive underwear versus pelvic floor muscle training or no treatment in women with symptoms of stress urinary incontinence: an assessor-blinded randomized control trial. Int Urogynecol J. (2019) 30(7):1093–9. 10.1007/s00192-018-03855-z 30627829

[17] Ninomiya S Saito I Masaki K Endo Y Morikawa S Okayama H. Single-Arm pilot study to determine the effectiveness of the support power of underwear in elevating the bladder neck and reducing symptoms of stress urinary incontinence in women. Low Urin Tract Symptoms. (2014) 6(2):81–7. 10.1111/luts.12023 26663545

[18] Szkwara JM Hing W Pope R Rathbone E. Compression shorts reduce prenatal pelvic and low back pain: a prospective quasi-experimental controlled study. PeerJ. (2019) 7:e7080. 10.7717/peerj.7080 31259096 PMC6589332

[19] Donnelly GM Liddle SD. Transperineal ultrasound imaging to investigate the mechanical influence of EVBTM sports compression shorts on bladder neck height in healthy adult female volunteers: a feasibility study. J Pelvic Obstet Gynaecol Physiother. (2025) 137(Autumn):24–37. 10.62399/MEPV7326

[20] Eldridge SM Chan CL Campbell MJ Bond CM Hopewell S Thabane L. Lancaster GA; PAFS consensus group. CONSORT 2010 statement: extension to randomised pilot and feasibility trials. Pilot Feasibility Stud. (2016) 2:64. 10.1186/s40814-016-0105-8 27965879 PMC5154046

[21] Baessler K O'Neill SM Maher CF Battistutta D. Australian Pelvic floor questionnaire: a validated interviewer-administered pelvic floor questionnaire for routine clinic and research. Int Urogynecol J Pelvic Floor Dysfunct. (2009) 20(2):149–58. 10.1007/s00192-008-0742-4 18958382

[22] Baessler K O'Neill SM Maher CF Battistutta D. A validated self-administered female pelvic floor questionnaire. Int Urogynecol J. (2010) 21(2):163–72. 10.1007/s00192-009-0997-4 19756341

[23] Laycock J Jerwood D. Pelvic floor muscle assessment: the PERFECT scheme. Physiotherapy. (2001) 87(12):631–42. 10.1016/S0031-9406(05)61108-X

[24] Swift S Morris S McKinnie V Freeman R Petri E Scotti RJ Validation of a simplified technique for using the POPQ pelvic organ prolapse classification system. Int Urogynecol J Pelvic Floor Dysfunct. (2006) 17(6):615–20. 10.1007/s00192-006-0076-z 16598414

[25] Parekh M Swift S Lemos N Iskander M Freeman B Arunkalaivanan AS Multicenter inter-examiner agreement trial for the validation of simplified POPQ system. Int Urogynecol J. (2011) 22(6):645–50. 10.1007/s00192-011-1395-2 21431391

[26] Persu C Chapple CR Cauni V Gutue S Geavlete P. Pelvic organ prolapse quantification system (POP-Q) - a new era in pelvic prolapse staging. J Med Life. (2011) 4(1):75–81. 21505577 PMC3056425

[27] Raizada N Mittal P Suri J Puri A Sharma V. Comparative study to evaluate the intersystem association and reliability between standard pelvic organ prolapse quantification system and simplified pelvic organ prolapse scoring system. J Obstet Gynaecol India. (2014) 64(6):421–4. 10.1007/s13224-014-0537-0 25489146 PMC4257927

[28] Beckley I Harris N. Pelvic organ prolapse: a urology perspective. J Clin Urol. (2013) 6(2):68–76. 10.1177/2051415812472675

[29] Besier TF Sturnieks DL Alderson JA Lloyd DG. Repeatability of gait data using a functional hip joint centre and a mean helical knee axis. J Biomech. (2003) 36(8):1159. 10.1016/s0021-9290(03)00087-3 12831742

[30] Reenalda J Maartens E Buurke JH Gruber AH. Kinematics and shock attenuation during a prolonged run on the athletic track as measured with inertial magnetic measurement units. Gait Posture. (2019) 68:155–60. 10.1016/j.gaitpost.2018.11.020 30481697

[31] Lindorfer J Kröll J Schwameder H. Familiarisation of novice and experienced treadmill users during a running session: group specific evidence, time and individual patterns. Hum Mov Sci. (2020) 69:102530. 10.1016/j.humov.2019.102530 31739233

[32] Oliveira AS Pirscoveanu CI. Implications of sample size and acquired number of steps to investigate running biomechanics. Sci Rep. (2021) 11(1):3083. 10.1038/s41598-021-82876-z 33542463 PMC7862397

[33] Osarogiagbon RU Vega DM Fashoyin-Aje L Wedam S Ison G Atienza S Wade JL 3rd. Modernizing clinical trial eligibility criteria: recommendations of the ASCO-friends of cancer research prior therapies work group. Clin Cancer Res. (2021 May 1) 27(9):2408. 10.1158/1078-0432.CCR-20-3854 33563637 PMC8170959

[34] AHCJ. Washout period. Medical Studies. (2025). Available online at: https://healthjournalism.org/glossary-terms/washout-period/ (Accessed February 6, 2025).

[35] Schwartz MH Rozumalski A. A new method for estimating joint parameters from motion data. J Biomech. (2005) 38(1):107–16. 10.1016/j.jbiomech.2004.03.009 15519345

[36] HAS-Motion. Functional Joints. Visual3d Documentation: Modeling. (2017). Available online at: https://wiki.has-motion.com/doku.php?id=visual3d:documentation:modeling:functional_joints:functional_joints (Accessed August 4, 2025).

[37] Winter DA. Biomechanics and Motor Control of Human Movement. Hoboken, NJ: John Wiley & Sons (2009).

[38] Moe-Nilssen R. A new method for evaluating motor control in gait under real-life environmental conditions. Part 1: the instrument. Clin Biomech (Bristol, Avon). (1998) 13(4–5):320–7. 10.1016/S0268-0033(98)00089-8 11415803

[39] Gruber AH Boyer KA Derrick TR Hamill J. Impact shock frequency components and attenuation in rearfoot and forefoot running. J Sport Health Sci. (2014) 3(2):113–21. 10.1016/j.jshs.2014.03.004

[40] Hunter JG Garcia GL Shim JK Miller RH. Fast running does not contribute more to cumulative load than slow running. Med Sci Sports Exerc. (2019) 51(6):1178–85. 10.1249/MSS.0000000000001888 30694982

[41] Giagio S Salvioli S Innocenti T Gava G Vecchiato M Pillastrini P PFD-SENTINEL: development of a screening tool for pelvic floor dysfunction in female athletes through an international delphi consensus. Br J Sports Med. (2023) 57(14):899–905. 10.1136/bjsports-2022-105985 36517214

[42] Skaik Y. The bread and butter of statistical analysis “*t*-test”: uses and misuses. Pak J Med Sci. (2015) 31(6):1558–9. 10.12669/pjms.316.8984 26870136 PMC4744321

[43] Chowdhury MZI Turin TC. Variable selection strategies and its importance in clinical prediction modelling. Fam Med Community Health. (2020) 8(1):e000262. 10.1136/fmch-2019-000262 32148735 PMC7032893

[44] Miller A Panneerselvam J Liu L. A review of regression and classification techniques for analysis of common and rare variants and gene-environmental factors. Neurocomputing. (2022) 489:466–85. 10.1016/j.neucom.2021.08.150

[45] Smith G. Step away from stepwise. J Big Data. (2018) 5(1):32. 10.1186/s40537-018-0143-6

[46] Hedberg EC Ayers S. The power of a paired *t*-test with a covariate. Soc Sci Res. (2015) 50:277–91. 10.1016/j.ssresearch.2014.12.004 25592936 PMC4297322

[47] Pataky TC. Generalized n-dimensional biomechanical field analysis using statistical parametric mapping. J Biomech. (2010) 43(10):1976–82. 10.1016/j.jbiomech.2010.03.008 20434726

[48] Brandt C. Physiotherapy and pelvic floor health within a contemporary biopsychosocial model of care: from research to education and clinical practice. S Afr J Physiother. (2021) 77(1):1538. 10.4102/sajp.v77i1.1538 34192209 PMC8182461

[49] Dasikan Z Ozturk R Ozturk A. Pelvic floor dysfunction symptoms and risk factors at the first year of postpartum women: a cross-sectional study. Contemp Nurse. (2020) 56(2):132–45. 10.1080/10376178.2020.1749099 32216721

[50] Proulx L LaCross J Lewis CL. Exploring the connection between the hip and pelvic floor in women with exercise-induced urinary incontinence: integrating clinical hypotheses with current evidence. J Pelvic Obstet Gynaecol Physiother. (2025) 137(Autumn):38–53. 10.62399/QFKG9942 PMC13574321 42741496

[51] Neumann DA. Kinesiology of the hip: a focus on muscular actions. J Orthop Sports Phys Ther. (2010) 40(2):82–94. 10.2519/jospt.2010.3025 20118525

[52] Broatch JR Brophy-Williams N Phillips EJ O'Bryan SJ Halson SL Barnes S Compression garments reduce muscle movement and activation during submaximal running. Med Sci Sports Exerc. (2020) 52(3):685–95. 10.1249/MSS.0000000000002182 31592978 PMC7034367

[53] Niederauer S Bérubé MÈ Brennan A McLean L Hitchcock R. Pelvic floor tissue damping during running using an intra-vaginal accelerometry approach. Clin Biomech (Bristol. (2022) 92:105554. 10.1016/j.clinbiomech.2021.105554 34974336 PMC8863648

[54] Gabbett TJ Oetter E. From tissue to system: what constitutes an appropriate response to loading? Sports Med. (2025) 55(1):17–35. 10.1007/s40279-024-02126-w 39527327

[55] Verhagen E Gabbett T. Load, capacity and health: critical pieces of the holistic performance puzzle. Br J Sports Med. (2019) 53(1):5–6. 10.1136/bjsports-2018-099819 30254049

[56] Kennaway B. Pelvic health physiotherapy for the management of pelvic floor dysfunction in the recreationally active female. Pelvic Obstetric and Gynaecological Physiotherapy. (2022) 131:33–41.

[57] Schache AG Blanch P Rath D Wrigley T Bennell K. Three-dimensional angular kinematics of the lumbar spine and pelvis during running. Hum Mov Sci. (2002) 21(2):273–93. 10.1016/s0167-9457(02)00080-5 12167303

[58] Seay JF Van Emmerik RE Hamill J. Low back pain status affects pelvis-trunk coordination and variability during walking and running. Clin Biomech (Bristol). (2011) 26(6):572–8. 10.1016/j.clinbiomech.2010.11.012 21536356

[59] Thein-Nissenbaum JM Thompson EF Chumanov ES Heiderscheit BC. Low back and hip pain in a postpartum runner: applying ultrasound imaging and running analysis. J Orthop Sports Phys Ther. (2012) 42(7):615. 10.2519/jospt.2012.3941 22446476

[60] Koenig I Eichelberger P Luginbuehl H Kuhn A Lehmann C Taeymans J Activation patterns of pelvic floor muscles in women with incontinence while running: a randomized controlled trial. Int Urogynecol J. (2021) 32(2):335–43. 10.1007/s00192-020-04334-0 32472161

[61] Constantinou CE Govan DE. Spatial distribution and timing of transmitted and reflexly generated urethral pressures in healthy women. J Urol. (1982) 127(5):964–9. 10.1016/S0022-5347(17)54148-8 7201031

[62] Moser H Leitner M Baeyens JP Radlinger L. Pelvic floor muscle activity during impact activities in continent and incontinent women: a systematic review. Int Urogynecol J. (2018) 29(2):179–96. 10.1007/s00192-017-3441-1 28884367

[63] Vleeming A Schuenke M. Form and force closure of the sacroiliac joints. PM R. (2019) 11(Suppl 1):S24–31. 10.1002/pmrj.12205 31218826

[64] Tawara D Nishiki T Ninomiya S Okayama H Naito K Morikawa S. Development of primary design guidelines for supportive underwear to elevate the bladder neck in women based on finite element analysis of the pelvis. Proc Inst Mech Eng H. (2022) 236(2):269–78. 10.1177/09544119211047058 34546113

[65] Gerges B Kamisan Atan I Shek KL Dietz HP. How to determine “ballooning” of the levator hiatus on clinical examination: a retrospective observational study. Int Urogynecol J. (2013) 24(11):1933–7. 10.1007/s00192-013-2119-6 23685723

[66] Berube ME Niederauer S Graham R Hitchcock R McLean L. Is pelvic floor loading in female runners associated with post-run changes in pelvic floor morphometry or function? BJU Int. (2025) 136(4):707–18. 10.1111/bju.16842 40586130 PMC12415321

[67] Bérubé M-È McLean L. The acute effects of running on pelvic floor morphology and function in runners with and without running-induced stress urinary incontinence. Int Urogynecol J. (2024) 35(1):127–38. 10.1007/s00192-023-05674-3 37991566 PMC10811036

[68] DeLancey JO Pipitone F Masteling M Xie B Ashton-Miller JA Chen L. Functional anatomy of urogenital hiatus closure: the perineal Complex triad hypothesis. Int Urogynecol J. (2024) 35(2):441–9. 10.1007/s00192-023-05708-w 38206338 PMC11060667

[69] DeLancey JOL Ashton-Miller JA LaCross J Pipitone F Schmidt P Chen L. Urogenital hiatus closure: facts, fallacies, and why a unified theory of hiatal failure is needed. Int Urogynecol J. (2025) 36(8):1581–9. 10.1007/s00192-025-06153-7 40332520 PMC12464070

[70] Mills C Loveridge A Milligan A Scurr J. Trunk marker sets and the subsequent calculation of trunk and breast kinematics during treadmill running. Text Res J. (2015) 86(11):1128–36. 10.1177/0040517515609257

