# Supplementary material for: Pelvic compression garments alter running biomechanics, perceived support, and fear of symptoms in postpartum women with pelvic floor dysfunction: preliminary observations from an exploratory, randomised, repeated-measures crossover design
Source: Front Sports Act Living. 2026 Jan 9;7:1691794. doi: 10.3389/fspor.2025.1691794 (PMC12827574; doi:10.3389/fspor.2025.1691794)
Supplement: Supplementary file 1 [file Supplementaryfile1.docx]

Appendix I – Study flow diagram for participant testing


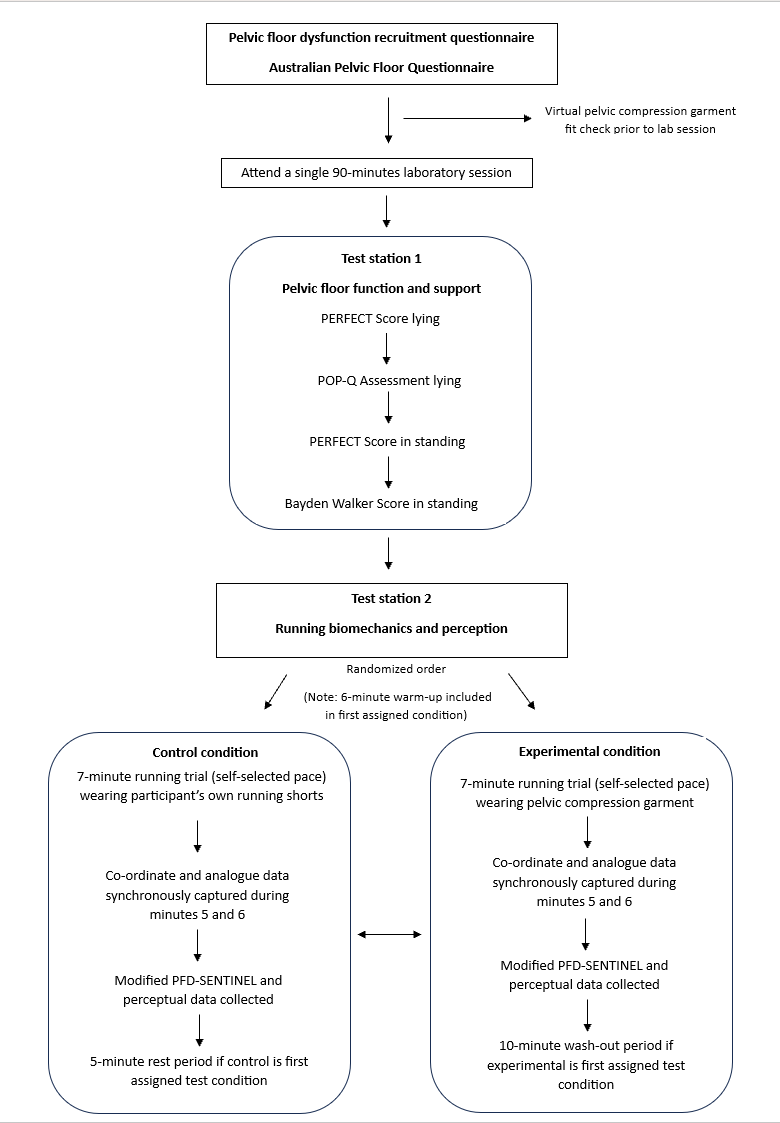


Virtual pelvic compression garment fit check prior to lab session

**Pelvic floor dysfunction recruitment questionnaire**

**Australian Pelvic Floor Questionnaire**

Attend a single 90-minutes laboratory session

**Test station 1**

**Pelvic floor function and support**

PERFECT Score lying

POP-Q Assessment lying

PERFECT Score in standing

Bayden Walker Score in standing

**Test station 2**

**Running biomechanics and perception**

7-minute running trial (self-selected pace) wearing participant’s own running shorts

**Control condition**

7-minute running trial (self-selected pace) wearing pelvic compression garment

Randomized order

(Note: 6-minute warm-up included in first assigned condition)

Co-ordinate and analogue data synchronously captured during minutes 5 and 6

Modified PFD-SENTINEL and perceptual data collected

5-minute rest period if control is first assigned test condition

Co-ordinate and analogue data synchronously captured during minutes 5 and 6

Modified PFD-SENTINEL and perceptual data collected

10-minute wash-out period if experimental is first assigned test condition

**Experimental condition**
