# Supplementary material for: Pelvic compression garments alter running biomechanics, perceived support, and fear of symptoms in postpartum women with pelvic floor dysfunction: preliminary observations from an exploratory, randomised, repeated-measures crossover design
Source: Front Sports Act Living. 2026 Jan 9;7:1691794. doi: 10.3389/fspor.2025.1691794 (PMC12827574; doi:10.3389/fspor.2025.1691794)
Supplement: Supplementary file 2 [file Supplementaryfile2.docx]

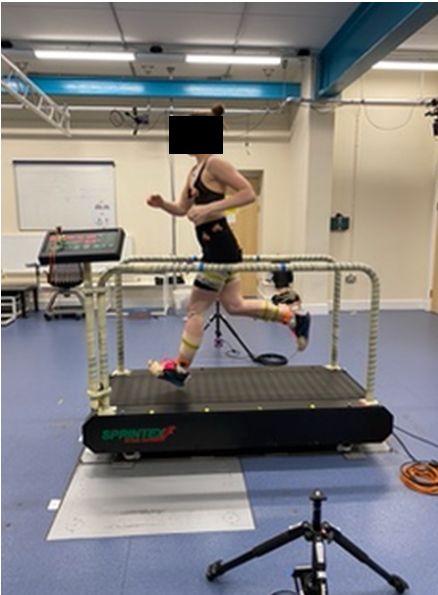


Figure 1 – Example participant on treadmill in the laboratory with motion camera system in place


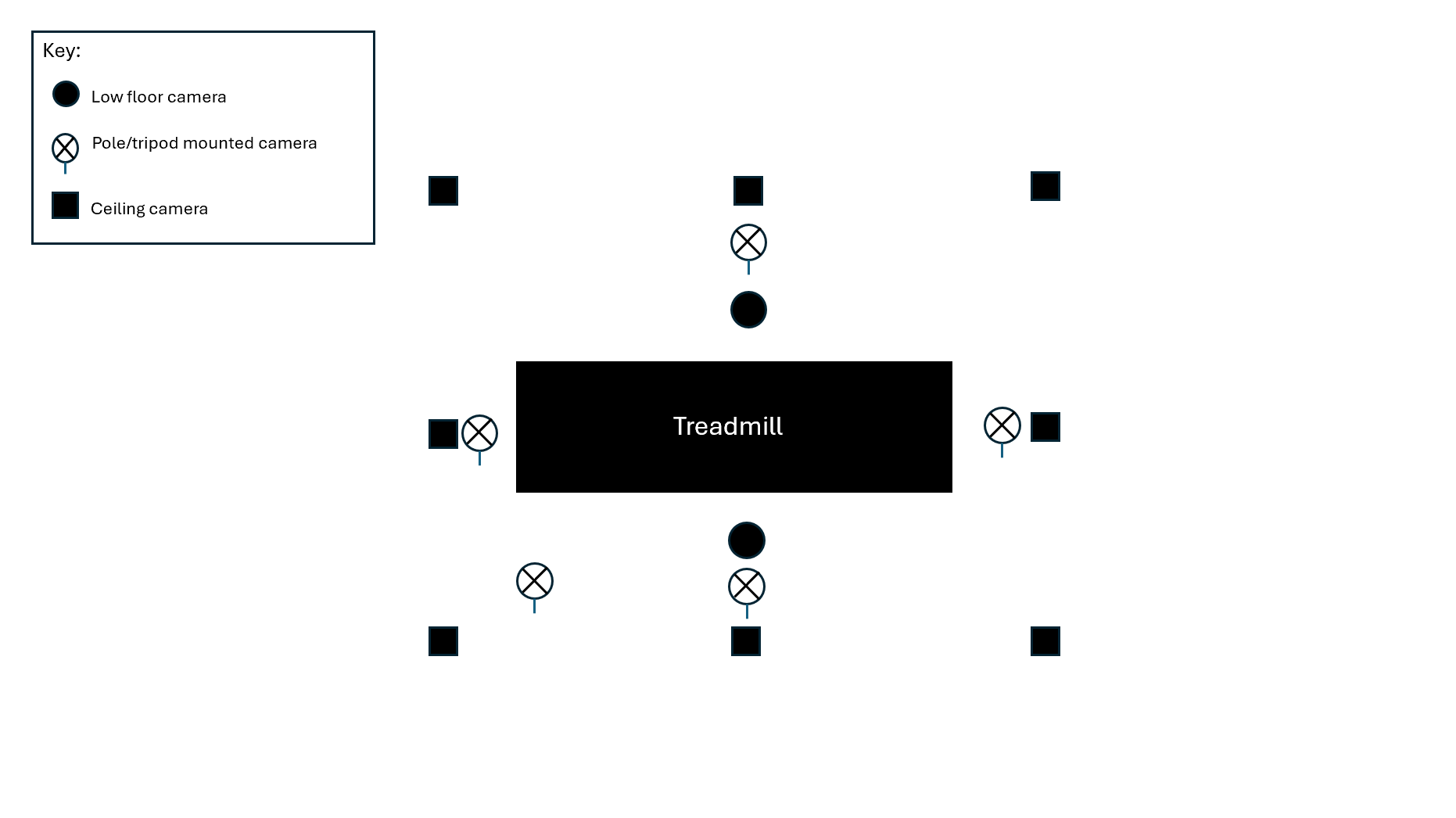


Figure 2 - schematic of 15-camera three-dimensional motion analysis system surrounding treadmill
