# Supplementary material for: Pelvic compression garments alter running biomechanics, perceived support, and fear of symptoms in postpartum women with pelvic floor dysfunction: preliminary observations from an exploratory, randomised, repeated-measures crossover design
Source: Front Sports Act Living. 2026 Jan 9;7:1691794. doi: 10.3389/fspor.2025.1691794 (PMC12827574; doi:10.3389/fspor.2025.1691794)

**Data distribution**

Example Q-Q Plots and histograms used for visual inspection of data distribution for individual variables during t-testing:


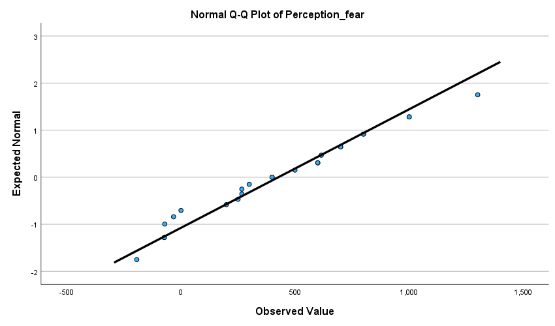

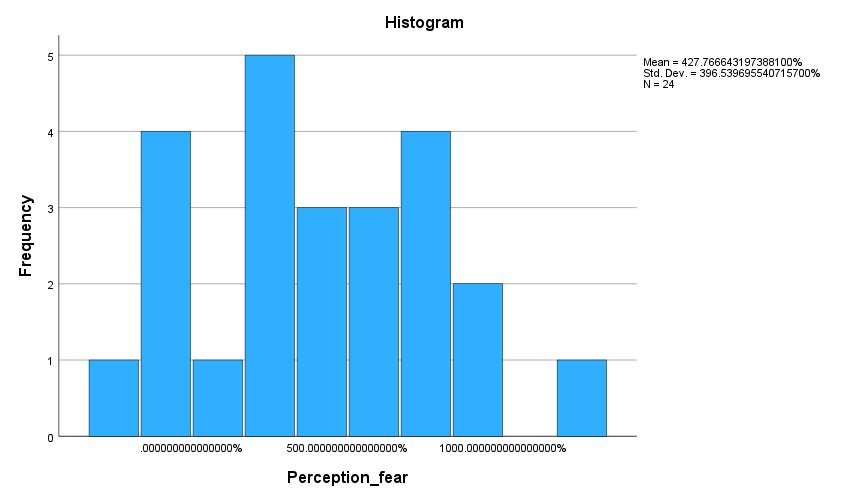


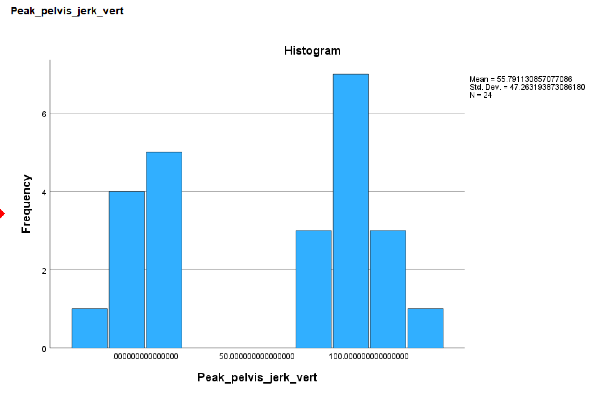


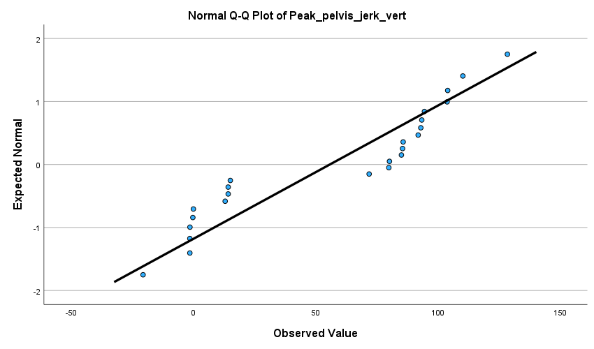


Example scatterplots used to visually inspect data distribution for individual variables during stepwise linear regression:


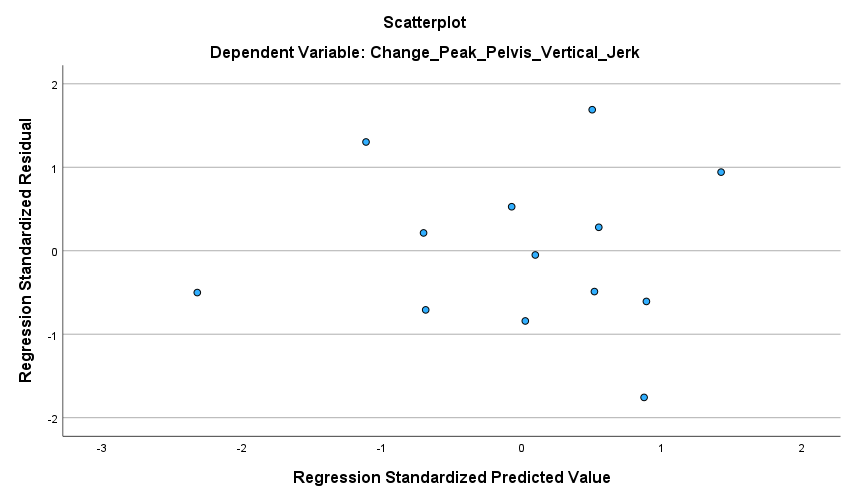


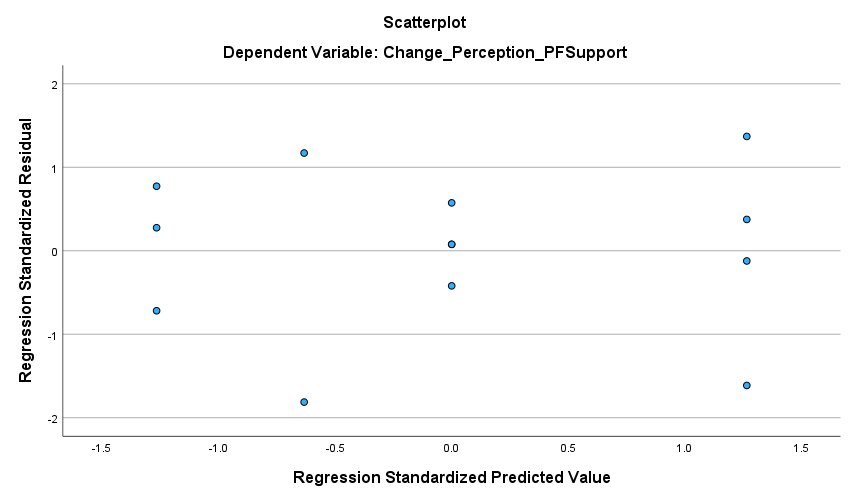

Supplement: Supplementary file 3 [file Supplementaryfile3.docx]
